# Amputation at the Shoulder Joint

**Published:** 1860-02

**Authors:** 


### NOTES OF SURGICAL CASES.

1. Amputation at the Shoulder Joint.—1. Peter M. Curdy, æt. 21 years, entered the Chicago City Hospital, Dec. 12, 1859, with a large tumor of the left arm. He stated that one year previously, in December, 1858, he had a dislocation of the left shoulder, which remained unreduced for eight weeks. From the time of reduction the shoulder remained painful, and in July, 1859, he perceived a hard tumor growing from the bone, about four inches below the shoulder joint. This increased in size rapidly, until his entrance into the hospital, when it measured 24½ inches in circumference. His general health was good, although the pain resulting from its weight have recently deprived him of sleep.

The operation was performed January, 4, 1860, in the follow- manner:—

An incision was commenced upon the top of the shoulder, and carried down upon the tumor, then curved forward and carried down to the axilla; a similar incision commencing at the first was carried down behind. Finding the end of the acromon process involved, this was sawn across. The anterior flap was then completed by cutting down into the axilla, and the artery tied immediately below the clavicle. The subclavian artery had been compressed where it passes over the first rib until this part of the operation was completed. The bone was disarticulated and the operation completed as soon as possible. The venous hæmorrhage was copious and six arteries required ligatures.

The patient bore the operation well, and for twelve days no symptoms of a dangerous character were noticed. During the night of the 16th of January, a copious hæmorrhage occurred which so reduced him, that he died about eight hours after.

On dissection the hæmorrhage was found to have occurred from the axillary artery where it was tied. No coagulum had formed within it in consequence of three arteries of considerable size originating near the point of ligature, and passing into the flaps in such a manner as to prevent the formation of an internal coagulum. The wound otherwise was of healthy appearance, and a large part of it had united by first intention and by cicatrization. The tumor seemed to be enchondroma.

2. This was the patient upon whom resection of the head of the humerus was performed November 27th, as reported in the number of this *Journal* for December, 1859.

The patient did well for five weeks, when an aneurismal tumor was perceived below the clavicle. This ruptured and a copious hæmorrhage occurred through the fistulous openings which still existed at the seat of injury. This was arrested in a degree by compression, and the amputation at the shoulder was performed in the same manner as in the preceding case. The loss of blood, however, had been too great, and the patient survived only about two days and a half.

